# A comparison between two types of limited sympathetic surgery for palmar hyperhidrosis

**DOI:** 10.1007/s00595-012-0246-1

**Published:** 2012-07-15

**Authors:** Jung Joo Hwang, Do Hyung Kim, Yoon Joo Hong, Doo Yun Lee

**Affiliations:** 1 Department of Thoracic and Cardiovascular Surgery, Eulji University Hospital, Daejeon, Korea; 2 Department of Thoracic and Cardiovascular Surgery, Eulji General Hospital, 280-1, Hagye-dong, Nowon-gu, Seoul, 139-711 Korea; 3 Department of Thoracic and Cardiovascular Surgery, GangNam Severenace Hospital, Seoul, Korea

**Keywords:** Palmar hyperhidrosis, Compensatory sweating

## Abstract

**Purpose:**

Endoscopic thoracic sympathetic surgery is effective for treating palmar hyperhidrosis, although compensatory sweating (CS) is a significant and annoying side effect. The purpose of this study was to compare the results of limited resection at two different locations.

**Methods:**

From May 2004 to June 2009, T3 sympathicotomy (group I) was performed in 46 patients and T3,4 ramicotomy (group II) was performed in 43 patients during the same period. T3 sympathicotomy (group I) and T3,4 ramicotomy (group II) were performed during the same period. Using questionnaires, completed by the patients, the satisfaction rates and grades of CS were analyzed.

**Results:**

No significant differences in age distribution or sex ratios were observed between the two groups. The satisfaction rate was 91.3 % in group I and 79.1 % in group II. The operation time was 19.8 (±6.6) min in group I, and 51.6 (±18.8) min in group II, showing a statistically significant difference (*p* < 0.002). The incidence of persistent hand sweating in group II (16.3 %) was higher than that observed in group I (2.2 %). The incidence of compensatory sweating on the lower extremities was higher in group II (37.2 %) than in group I (10.9 %).

**Conclusions:**

Although ramicotomy is considered to be an effective method for treating hyperhidrosis and has a theoretical advantage of allowing greater anatomical resection, it requires longer operation time and induces more severe compensatory sweating in the lower limbs resulting in reduced satisfaction rates.

## Introduction

Thoracoscopic sympathetic surgery is currently the most effective treatment for palmar hyperhidrosis, and the success rate of this procedure is quite high. However, the incidence of postoperative compensatory sweating (CS), which is the most common cause of patient dissatisfaction, has been reported to be as high as 67–89 % [1, 2]. To reduce CS, surgeons now attempt to limit the extent of sympathetic nerve resection [3–6].

In this study, we performed either sympathicotomy (to transect sympathetic nerves along the margin of the ribs) or ramicotomy (to cut only rami communicantes). Although ramicotomy is associated with a lower incidence of CS, the failure rate is approximately 30 % [4, 5]. The operative method was modified to cut two levels of rami communicantes (T3,4 ramicotomy) to reduce persistent sweating. Sympathicotomy was performed at one level, the lower margin of the third rib, for the purpose of preserving the T2 ganglion.

The aim of this study was to compare the efficiency of two-level ramicotomies with that of T3 sympathicotomy, which is the more widely accepted surgical method for treating palmar hyperhidrosis.

## Patients and methods

From March 2004 to June 2009, 89 patients with palmar hyperhidrosis underwent two different types of surgery during the same period. Plantar hyperhidrosis was combined in 45 patients (50.6 %), and the patients with combined facial or axillary hyperhidrosis and secondary hyperhidrosis were excluded from this study. The operations were performed by the same surgical team during this period. The operation method was determined according to the patient’s own decision after providing information regarding the two methods. Written informed consent was obtained from all patients before surgery. This study was approved by the IRB (institutional review board) of our hospital.

The patients received follow-up on an outpatient basis within 1 month of surgery and after 1 year using a survey via e-mail questionnaire or phone calls. The data were collected prospectively. The key components of the questionnaire inquired about the dryness of hands (graded as three categories: dry, humid or persistent) and the severity of compensatory sweating (classified into four categories: absent, mild, embarrassing or disabling, which meant that patients needed to change clothes during the day). We also evaluated the rates of satisfaction and the sites of compensatory sweating.

A review of the medical records was performed. Group I consisted of 46 patients who underwent T3 sympathicotomy to cut the thoracic sympathetic nerve at the lower margin of the third rib and group II consisted of 43 patients who underwent T3,4 ramicotomy to cut the rami communicantes of the T3 and T4 sympathetic nerves.

The data were analyzed with PASW 18.0 statistical software (SPSS Inc., Chicago, IL, USA), and probability values of less than 0.05 were considered to be significant. The distributions of continuous variables were expressed as the mean (±the standard deviation). Categorical variables were analyzed using the χ^2^ test and continuous data were analyzed using Student’s *t* test.

### Operation techniques

The patients were placed in a semi-Fowler’s position with both arms extended and the forearm abducted to 80° after establishing general anesthesia using a single lumen endotracheal tube. For each patient, the head was elevated for the purpose of pulling the lungs away from the apex. One port was created to introduce the thoracoscope through the sixth intercostal space on the anterior axillary line. Another port was created for the insertion of an endoscopic instrument through the forth intercostal space on the mid-axillary line using the same method described previously [5]. Performing T3,4 ramicotomy, the parietal pleura were opened along the main sympathetic trunk using electrical scissors. The sympathetic chain was dissected using endoscopic scissors with the least possible usage of electrocauterization to avoid any diathermic injury. After dissecting and dividing the T3 and T4 rami communicantes, using endoscopic scissors, the main trunk was lifted upward to observe the presence of any residual branches. The same procedures were performed on the contralateral side.

For T3 sympathicotomy, on the other hand, the chain was ablated with an electrical diathermy on the lower border of the third rib and was extended approximately 2 cm laterally for the purpose of ablating accessory nerve fibers. The operative procedures were completed after confirming the absence of intrathoracic bleeding and eliminating the air from the thoracic cavity using the CVP measuring catheters inserted through the troca along the guide wire and connected to the modified underwater seal device. No drainage tubes remained after surgery.

## Results

No statistically significant differences in age or sex distribution were observed between the two groups (Table 1). All operations were successfully performed without converting to thoracotomy. The mean operation time was 19.8 (±6.6) minutes in group I and 51.6 (±18.8) minutes in group II (*p* value <0.002). No operative mortalities occurred. No major complications, such as Horner syndrome or pulmonary edema, occurred. Residual pneumothorax was found on follow-up chest X-ray in three patients, and the condition resolved spontaneously in all cases. Minimal amounts of hemothorax were observed in one case in group II and were relieved by tube drainage.

**Table 1:** Patient demography

	Group I (%)	Group II (%)	*p* value
Sex			0.753
Male	24 (52.2)	21 (48.8)	
Female	22 (47.8)	22 (51.2)	
Plantar hypherhidrosis	21 (45.7)	24 (55.8)	0.228
Age	23.6 ± 7.2	24.7 ± 9.0	0.519
Complication	1 (2.1)	3 (7.0)	
Operation time (min)	19.8 ± 6.6	51.6 ± 18.8	0.002

Group I sympathicotomy group, Group II ramicotomy group)

Compensatory sweating occurred in 80.4 % of the patients in group I (sympathicotomy group) and 95.3 % of the patients in group II (ramicotomy group). Disabling sweating was found in 8.7 % of the patients in group I and 14.0 % of the patients in group II. Compensatory sweating occurred most frequently on the trunk in 67.4 % of the patients in group I and 76.7 % of the patients in group II. The incidence of excessive sweating on the lower limbs after surgery was different between the two groups (Table 2). 16 patients (37.2 %) in group II felt increased sweating on the sole and thigh areas; however, in group I, five patients (10.9 %, *p* value <0.003) reported excessive sweating on the soles and three patients reported excessive sweating in the groin and thigh areas(6.5 %, *p* value <0.001).

**Table 2:** Compensatory sweating after sympathetic surgery

Compensatory sweating	Group I		Group II		*p* value
	*N*	%	*N*	%	
Sweating					0.057
Absence	9	19.6	2	4.7	
Presence	37	80.4	41	95.3	
Location					
Face	5	10.9	2	4.7	0.436
Trunk	31	67.4	33	76.7	0.355
Groin and Thigh	3	6.5	16	37.2	0.001
Foot	5	10.9	16	37.2	0.003
Gustatory	12	26.1	14	32.6	0.641
Degree					
Absence	9	19.6	2	4.7	
Mild	17	37	21	48.8	
Embarrassing	16	34.8	14	32.6	
Disabling	4	8.7	6	14	

The satisfaction rates were 91.3 and 79.1 % in groups I and II, respectively. The major reason for dissatisfaction was compensatory sweating in both groups. Four patients (9.3 %) in group II experienced plantar sweating that directly contributed to postoperative dissatisfaction. However, the number of patients with combined plantar sweating prior to surgery was 21 (45.7 %) in group I and 24 (55.8%) in group II (*p* < 0.228).

The incidence of palmar dryness after surgery was also different between the two groups. Dry hands without any sweating were found in 82.6 % of patients who underwent sympathicotomy and 25.6 % of patients who underwent ramicotomy. Palmar humidity was maintained in 58.5 % of the patients in group II and 15.2 % of the patients in group I (*p* value <0.001) (Table 3). Persistent sweating defined as resweating within 1 year after surgery was observed in seven patients in group II and in only one patient in group I.

**Table 3:** Patient satisfaction and dryness after surgery

	Group I		Group II		*p* value
	*N*	%	*N*	%	
Satisfaction					0.067
Very satisfied	9	19.6	3	7	
Satisfied	33	71.7	31	72.1	
Dissatisfied	4	8.7	9	20.9	
Dryness					0.001
Dry	38	82.6	11	25.6	
Humid	7	15.2	25	58.1	
Persistent	1	2.2	7	16.3	

## Discussion

Thoracoscopic sympathetic surgery is currently accepted to be the most effective and endurable therapeutic method for treating palmar hyperhidrosis. However, postoperative compensatory sweating (CS) after thoracoscopic sympathetic surgery remains a major issue.

The common hypothesis regarding CS suggests that this phenomenon involves a thermoregulatory mechanism in which the sweat glands compensate for decreased amounts of secretory tissues after sympathectomy [7]. It has been reported that the total amount of body perspiration is not altered after sympathetic surgery, despite a reduction in hand sweat [8]. Therefore, it is suggested that CS involves a redistribution of sweat from areas denervated by surgery to uninjured areas under sympathetic control. This hypothesis is simple; however, it does not explain the wide variation of CS. Another hypothesis is that CS is caused by a feedback mechanism in the hypothalamus. Afferent thermal information from different body parts is transmitted to the hypothalamus, which releases efferent signals to the sweat glands. Interruption of the sympathetic ganglia blocks negative feedback signals and amplifies efferent signals. These signals induce excessive sweat on the body except in sympathetically denervated areas [9]. No theory can fully explain CS. However, based on these theories and the results of anatomical studies, various surgical methods have been attempted to manage CS.

One method is selective interruption of rami communicantes with preservation of the main trunk, first proposed and practiced by Wittmoser [10]. This method by far causes the least interference with the sympathetic system. It does lower the incidence of compensatory sweating; however, it is associated with a higher incidence of recurrence than conventional resection. Approximately 5.0–23.5 % of patients who undergo this procedure experience persistent sweating on one or both hands, which prevents it from being widely accepted. However, Gossot et al. [3] compared a group of patients who underwent T2–T4 sympathectomy with a group of patients who underwent T2–T4 ramicotomy. No differences in the incidence of CS were noted, although the incidence of severe CS was higher in the sympathectomy group (27 vs. 13 %). The authors concluded that preservation of the sympathetic trunk is important to decrease the incidence of CS.

In patients with facial hyperhidrosis, T2 ramicotomy has been compared with T2 clipping, [11] and as expected, the incidence of CS is lower in patients who undergo T2 ramicotomy and the rate of operation failure is higher compared to T2 clipping. Other studies have compared two-level ramicotomy (T2–T3) and single level ramicotomy (T3) with sympathectomy [12]. In those studies, CS occurred less frequently in the patients who underwent ramicotomy; however, the incidence of persistent postoperative sweating was relatively high.

Based on those results and the fact that T3,4 preganglionic fibers are considered to be the main lesions of hand sweating [13], we had adopted T3,4 ramicotomy to reduce the incidence and recurrence of CS. This method reduced the recurrence rate and increased the satisfaction rate compared to previous ramicotomy data (Table 4) [3–5, 12, 14].

**Table 4:** Operative results of ramicotomy reported in the literature [3–5, 12, 14]

Series	Number	Method	CS (%)	Satisfaction (%)	PS or recurrence (%)
Gossot [3]	62	T2–T4 or T5 ramicotomy	70.9	Not mentioned	10
Cho [12]	50	T2 and T3 ramicotomy	92	78	6
Lee [4]	68	T3 ramicotomy	67.4	67.6	32.5
Cho [14]	11	T3 or T4 ramicotomy	90.1	6.5 (10 scale)	21.4
Kim [11]	83	T3 ramicotomy	62.1	68.6	30.1
Present study	43	T3 and T4 ramicotomy	95.3	79.1	16.3

*Number* number of patients, *CS* compensatory sweating, *PS* persistent sweating

Limited sympathicotomy is another method of reducing CS with a high success rate. During the last two decades, we also changed our strategy of treating palmar hyperhidrosis from sympathectomy (resection of sympathetic ganglion) to sympathicotomy to reduce CS and to preserve the T2 ganglion. It is known that preserving the T2 ganglion and performing resection more caudal to the T2 ganglion reduces CS [9, 15]. In our study, we adopted T3 sympathicotomy. T3 sympathicotomy requires shorter operation times and induces less injury to the pleura. Therefore, the incidence of complications is lower than that observed with ramicotomy. In this study, the patients who underwent sympathicotomy had better outcomes than those who underwent ramicotomy(Table 5) [6, 16, 17]. Recently, T4 sympathicotomy has been reported to be superior to T3 sympathicotomy, because it results in reduced CS and only slight hand moisture. The results are felt to be more natural by patients. In this study, we compared only T3 sympathicotomy patients to ramicotomy patients because we had recently adopted T4 sympathicotomy. Therefore, the number of cases available for comparison was small.

**Table 5:** Operative results of sympathicotomy reported in the literature [6, 16, 17]

Series	Number	Method	CS (%)	Satisfaction (%)	PS or recurrence (%)
Liu [6]	68	T3 sympathicotomy	77.4	95.1	Not mentioned
	73	T4 sympathicotomy	56.5	94.2	Not mentioned
Kim [16]	56	T3 sympathicotomy	82.1	85.7	1.8
	63	T4 sympathicotomy	17.5	92.1	3.2
Chang [17]	86	T2 sympathectomy	92	6.3 (10 scale)	25.6
	78	T3 sympathectomy	92	7.8 (10 scale)	7.7
	70	T4 sympathectomy	80	7.9 (10 scale)	22.9
Present study	46	T3 sympathicotomy	80.4	91.3	2.2

*Number* number of patients, *CS* compensatory sweating, *PS* persistent sweating

More than 80 % of the patients with palmar hyperhidrosis had associated plantar hyperhidrosis. Hsu et al. [18]. reported that 64 % of patients with plantar hyperhidrosis are cured after undergoing T2 sympathectomy. Wolosker et al. [19] reported that 62.5 % of patients improve after 1 year. As time progresses, the satisfaction rates decrease. It is supposed that the worsening of plantar hyperhidrosis over time may be related to a return of the emotional stress that was initially lessened through the high degree of satisfaction obtained from the postoperative palmar anhidrosis. In these cases, no actual worsening of plantar sweating occurred. However, in our cases, compared with the T3 sympathicotomy group, the main reason for CS in the ramicotomy group was aggravated sweat on the lower legs. Increased sweat on the lower extremities after ramicotomy directly affected dissatisfaction in four patients (9.3 %) in our data.

Cho et al. [12] reported that lower extremity sweating occurs in 28 % of ramicotomy cases on the presumption that ramicotomy leaves the main trunk intact without disrupting sympathetic chains, while sympathicotomy interrupt sympathetic chains. Ironically, ramicotomy affects sweating on the soles more sensitively than the palms because feedback pathways are not interrupted. This might explain why more aggravated plantar sweating was observed in the ramicotomy group than in sympathicotomy group.

In conclusion, despite the potential advantages of treating hyperhidrosis with anatomic resection to provide natural postoperative conditions of adequately humid hands, ramicotomy shows two clear limitations: first, the two-level ramicotomy method results in a higher recurrence rate than T3 sympathicotomy and second, unexpected excessive sweating occurs on the lower extremities. Although the exact mechanisms have not been established, we suppose that this phenomenon is related to the preservation of the main sympathetic trunk. The unexpected lower extremity sweating that occurs following ramicotomy leads to lower levels of satisfaction compared with sympathicotomy.

## References

[1] Reisfeld R, Nguyen R, Pnini A (2000). Endoscopic thoracic sympathectomy for treatment of essential hyperhidrosis syndrome: experience with 650 patients. Surg Laparosc Endosc Percutan Tech.

[2] Gossot D, Galetta D, Pascal A, Debrosse D, Caliandro R, Girard P, Stern JB, Grunenwald D (2003). Long-term results of endoscopic thoracic sympathectomy for upper limb hyperhidrosis. Ann Thorac Surg.

[3] Gossot D, Toledo L, Fritsch S (1997). Thoracoscopic sympathectomy for upper limb hyperhidrosis: looking for the right operation. Ann Thorac Surg.

[4] Lee DY, Paik HC, Kim DH, Kim HW. Comparative analysis of T3 selective division of rami communicantes (ramicotomy) to T3 sympathetic clipping in treatment of palmar hyperhidrosis. Clin Auton Res. 2003;13 Suppl 1:145–7. 10.1007/s10286-003-1115-1 14673673

[5] Lee DY, Kim DH, Paik HC (2004). Selective division of T3 rami communicantes (T3 ramicotomy) in the treatment of palmar hyperhidrosis. Ann Thorac Surg.

[6] Liu Y, Yang J, Yang F, Liang G, Li J, Huang Y, Wang J (2009). Surgical treatment of primary palmar hyperhidrosis: a prospective randomized study comparing T3 and T4 sympathicotomy. Eur J Cardiothorac Surg.

[7] Shelley WB, Florence R (1960). Compensatory hyperhidrosis after sympathectomy. N Eng J Med.

[8] Shoenfeld Y, Shapiro Y, Machtiger A, Magazanik A (1976). Sweat studies in hyperhidrosis palmaris and plantaris. A survey of 60 patients before and after cervical sympathectomy. Dermatologica.

[9] Lin CC, Telaranta T (2001). Lin-Telaranta classification: the importance of different procedures for different indications in sympathetic surgery. Ann Chir Gynaecol.

[10] Wittmoser R, Cushieri A, Buess G, Perissat J (1992). Thoracoscopic sympathectomy and vagotomy. Operative manual of endoscopic surgery.

[11] Kim DH, Paik HC, Lee DY (2004). Comparative analysis of T2 selective division of rami-communicantes (ramicotomy) with T2 sympathetic clipping in the treatment of craniofacial hyperhidrosis. Eur J Cardiothorac Surg.

[12] Cho HM, Paik HC, Kim DH, Ham SJ, Lee DY (2002). Ramicotomy of T2,3 sympathetic ganglia for palmar hyperhidrosis. Korean J Thorac Cardiovasc Surg.

[13] Gray H. The sympathetic nerves. In: Lewis WH, editor. Anatomy of the human body. 20th ed., Philadelphia: Lea & Febiger; 2000. pp. 1292–9.

[14] Cho HM, Chung KY, Kim DJ, Lee KJ, Kim KD (2003). The comparison of VATS ramicotomy and VATS sympathicotomy for treating essential hyperhidrosis. Yonsei Med J.

[15] Chou SH, Kao EL, Lin CC, Chang YT, Huang MF (2006). The importance of classification in sympathetic surgery and a proposed mechanism for compensatory hyperhidrosis: experience with 464 cased. Surg Endosc.

[16] Kim WO, Kil HK, Yoon KB, Yoon DM, Lee JS (2010). Influence of T3 or T4 sympathicotomy for palmar hyperhidrosis. Am J Surg.

[17] Chang YT, Li HP, Lee JY, Lin PJ, Lin CC, Kao EL, Chou SH, Huang MF (2007). Treatment of palmar hyperhidrosis: T(4)level compared with T(3) and T(2). Ann Surg.

[18] Hsu CP, Chen CY, Lin CT, Wang JH, Chen CL, Wang PY (1995). Video-assisted thoracoscopic T2 sympathectomy for hyperhidrosis palmaris. J Am Coll Surg.

[19] Wolosker N, Yazbek G, Milanez de Campos JR, Kauffman P, Ishy A, Puech-Leão P (2007). Evaluation of plantar hyperhidrosis in patients undergoing video-assisted thoracoscopic sympathectomy. Clin Auton Res.

